# Supplementary material for: The impact of heating, ventilation, and air conditioning design features on the transmission of viruses, including the 2019 novel coronavirus: A systematic review of ventilation and coronavirus
Source: PLOS Glob Public Health. 2022 Jul 5;2(7):e0000552. doi: 10.1371/journal.pgph.0000552 (PMC10021902; doi:10.1371/journal.pgph.0000552)
Supplement: Attachment — Submitted filename: Ventilation-Response to Reviewer Comments 2022apr23.docx [file pgph.0000552.s002.docx]

**Manuscript ID**: PGPH-D-21-00860

**Title:** The impact of heating, ventilation, and air conditioning design features on the transmission of viruses, including the 2019 novel coronavirus: a systematic review of ventilation and coronavirus

Dear Dr. Adetokunboh,

Thank you for the opportunity to respond to your comments and the reviewer comments and submit a revised version of our manuscript. We have provided a response to each comment below. We have uploaded a marked-up copy of our manuscript showing the changes, as well as an unmarked copy of the revised manuscript. We thank you for your time and consideration and look forward to your response.

Best regards,

Brian Fleck Ph.D.,P.Eng.,ICD.D.
Professor, Mechanical Engineering, UAlberta

**Additional Editor Comments (if provided):**

This is a well written systematic review on a very important and critical subject. However, find below few comments:

*Authors’ response: Thank you for this positive comment. We have addressed all of the detailed comments below.*

1. Page 2; Line 20: Write the official name of SARS-CoV-2 first.

*Authors’ response: We have added “Severe Acute Respiratory Syndrome coronavirus 2” to the Abstract.*

2. Page 4; Line 47: Change "[a]irborne".

*Authors’ response: We have replaced with an upper-case “A” consistent with the original source for the quotation.*

3. Page 10; Line 166: Write out carbon dioxide in full first.

*Authors’ response: We have added “carbon dioxide” as suggested. Thank you for catching this oversight.*

4. Include the list of abbreviations.

*Authors’ response: We have added a list of abbreviations used in the text of the manuscript on page 38. Abbreviations used in tables appear as footnotes to the relevant table.*

5. State the strengths and limitations of this study.

*Authors’ response: We have added a “Strengths and Limitations” section on page 37.*

6. Please state the role of each reviewer.

*Authors’ response: We have added authors’ contributions on page 40.*

7. References No 1: The link is inactive.

*Authors’ response: we have confirmed that the website in Reference No. 1 is active:* https://www.ashrae.org/about/news/2021/ashrae-epidemic-task-force-releases-updated-airborne-transmission-guidance

8. Figure 1: The title should be underneath the flow chart.

*Authors’ response: The title was moved to appear underneath the figure.*

**Review Comments to the Author**

Reviewer #2: Title: The impact of heating, ventilation, and air conditioning design features on the transmission of viruses, including the 2019 novel coronavirus: a systematic review of ventilation and coronavirus

Comment: Generally, the study is good to be published, however, it needs some correction. The manuscript has not taken on board the title. When reading the manuscript, you don’t find the issues of heating and air conditioning being mentioned.

Suggestion: The title should read as ‘The impact of heating, ventilation, and air conditioning design features on the transmission of viruses, including the 2019 novel coronavirus: a systematic review of ventilation and coronavirus and then the rest of the manuscript should remain as it is OR the manuscript should be rewritten to take on board the issues of heating and air conditioning design features.

*Authors’ response: The suggested title is the same as the existing title. We have elected to keep the title as-is. The search terms, as detailed in Table 1, were comprehensive to ensure that we retrieved all of the available articles on the design features for heating, ventilation and air conditioning. Our paper discusses the articles retrieved from this comprehensive search and we have elected to keep the title the same.*

Reviewer #3: This manuscript is a very well written and clear systematic review of the effectiveness of ventilation for mitigating transmission of coronavirus. I agree with the conclusions made from the analysis and found no grammatical errors. An enjoyable read of important evidence synthesis. No further comments.

*Authors’ response: Thank you for these positive comments. No changes required.*

Reviewer #4: The authors have produced a very thorough and well written systematic review of the literature on how HVAC systems influence coronavirus transmission. The analysis and findings are clearly presented and the discussion highlights gaps in the literature which require future research.

*Authors’ response: Thank you for these positive comments. No changes required.*

Reviewer #5: A useful summary of the currently available information. I have no specific comments or suggestions for improvement or clarity. Looking forward to the paper's publication and the discussion it generates.

*Authors’ response: Thank you for these positive comments. No changes required. We also look forward to future discussion on this important topic.*
